# Identification of novel compound heterozygous mutations in *ACO2* in a patient with progressive cerebral and cerebellar atrophy

**DOI:** 10.1002/mgg3.698

**Published:** 2019-05-20

**Authors:** Masahide Fukada, Keitaro Yamada, Shima Eda, Ken Inoue, Chihiro Ohba, Naomichi Matsumoto, Hirotomo Saitsu, Atsuo Nakayama

**Affiliations:** ^1^ Department of Embryology Institute for Developmental Research, Aichi Human Service Center Kasugai Japan; ^2^ Department of Pediatric Neurology Aichi Prefectural Colony Central Hospital, Aichi Human Service Center Kasugai Japan; ^3^ Department of Mental Retardation and Birth Defect Research National Center of Neurology and Psychiatry Kodaira, Tokyo Japan; ^4^ Department of Human Genetics Yokohama City University Graduate School of Medicine Yokohama Japan; ^5^ Department of Biochemistry Hamamatsu University School of Medicine Hamamatsu Japan; ^6^ Department of Neurochemistry Nagoya University Graduate School of Medicine Nagoya Japan

**Keywords:** ACO2, aconitase, TCA cycle, mitochondria, brain atrophy, ataxia, hypotonia

## Abstract

**Background:**

The tricarboxylic acid (TCA) cycle is a sequence of catabolic reactions within the mitochondrial matrix, and is a central pathway for cellular energy metabolism. Genetic defects affecting the TCA cycle are known to cause severe multisystem disorders.

**Methods:**

We performed whole exome sequencing of genomic DNA of a patient with progressive cerebellar and cerebral atrophy, hypotonia, ataxia, seizure disorder, developmental delay, ophthalmological abnormalities and hearing loss. We also performed biochemical studies using patient fibroblasts.

**Results:**

We identified new compound heterozygous mutations (c.1534G > A, p.Asp512Asn and c.1997G > C, p.Gly666Ala) in *ACO2,* which encodes aconitase 2, a component of the TCA cycle. In patient fibroblasts, the aconitase activity was reduced to 15% of that of the control, and the aconitase 2 level decreased to 36% of that of the control. As such a decrease in aconitase 2 in patient fibroblasts was partially restored by proteasome inhibition, mutant aconitase 2 was suggested to be relatively unstable and rapidly degraded after being synthesized. In addition, the activity of the father‐derived variant of aconitase 2 (p.Gly666Ala), which had a mutation near the active center, was 55% of that of wild‐type.

**Conclusion:**

The marked reduction of aconitase activity in patient fibroblasts was due to the combination of decreased aconitase 2 amount and activity due to mutations. Reduced aconitase activity directly suppresses the TCA cycle, resulting in mitochondrial dysfunction, which may lead to symptoms similar to those observed in mitochondrial diseases.

## 1 INTRODUCTION

The tricarboxylic acid (TCA) cycle plays a central role in cellular energy metabolism in the mitochondrial matrix. Nicotinamide adenine dinucleotide (NADH) and flavin adenine dinucleotide (FADH_2_) produced by this cycle supply electrons for respiration, which results in efficient production of ATP. In addition, several intermediates in the TCA cycle are essential precursors of heme and some amino acids. Therefore, deficiency in the enzymes constituting the TCA cycle causes multisystem disorders, including neurodegenerative diseases and cancers (Wallace, Fan, & Procaccio, 2010; Raimundo, Baysal, & Shadel, 2011).

Aconitase (EC 4.2.1.3) is a TCA cycle enzyme that catalyzes the reversible isomerization of citrate to isocitrate via a cis‐aconitate intermediate. Humans have two aconitase isoforms, aconitase 1 and 2; only aconitase 2 acts as the TCA cycle enzyme in the mitochondrial matrix (Slaughter, Hopkinson, & Harris, 1977). Human aconitase 2, composed of 780 amino acids, is a monomeric enzyme (Slaughter, Hopkinson, & Harris, 1975) that is encoded by *ACO2* (OMIM 100850) on chromosome 22. Spiegel et al. first reported a pathogenic *ACO2* mutation (homozygous, p.Ser112Arg) in eight individuals from two unrelated Arab families, characterized by infantile cerebellar‐retinal degeneration. In addition to cerebellar atrophy and ophthalmological abnormalities, the patients also exhibited hypotonia, ataxia, seizure disorder, developmental delay, intellectual disability and hearing loss (Spiegel et al., 2012). Thereafter, one homozygous (p.Gly259Asp) and five compound heterozygous (p.[Lys736Asn;Lys776Asn], p.[Arg607Cys; Pro712Leu], p.[Gly620Asp;Gly683Val], p.[Val364Ala; Lys776Asnfs*49] and p.[Pro712Leu; Gly279_Glu313del]) mutations of *ACO2* were found in patients with similar clinical characteristics to those described by Spiegel (p.Ser112Arg), and one compound heterozygous mutation (p.[Leu74Val; Gly661Val]) in siblings having only ophthalmological abnormalities (Abela et al., 2017; Marelli et al., 2018; Metodiev et al., 2014; Sadat et al., 2016; Srivastava et al., 2017). Significant decreases in aconitase activity in patient cells were observed in most cases, and the impaired energy metabolism due to TCA cycle dysfunction was considered to be a major cause of symptoms in *ACO2* deficiency.

Here, we describe novel compound heterozygous missense mutations of *ACO2* found in a Japanese girl. The patient harboring these mutations exhibits most of the symptoms of *ACO2* deficiency reported thus far. In this study, we examined the effects of these mutations on enzymatic activity and stability.

## 2 MATERIALS AND METHODS

### 2.1 Ethics statement

This study was approved by the Institutional Review Board of Yokohama City University School of Medicine and Aichi Human Service Center (permission number: L17‐01). The parents of the patient gave informed consent for participation in this study.

### 2.2 Whole exome sequencing

Genomic DNA of the patient was extracted using the SureSelect Human All Exon v5 kit (Agilent Technologies, Santa Clara, CA) and sequenced on the HiSeq 2000 (Illumina, San Diego, CA) with 101‐bp paired end reads. Exome data processing, variant calling and variant annotation were performed as previously described (Fukai et al., 2016). The mean read depth of the protein‐coding regions of RefSeq genes was 127.75, and 95.1% of the targeted coding sequences were covered by 20 reads or more. We focused on rare nonsynonymous variants showing a minor allele frequency below 1% in both dbSNP135 (http://www.ncbi.nlm.nih.gov/SNP/) and our in‐house 575 control exomes. In this report, *ACO2* mutations are described with reference to the *ACO2* mRNA sequence (GenBank accession number, NM_001098.3).

### 2.3 Cell culture, DNA construction, aconitase assay, immunofluorescence and Western blotting

Skin fibroblasts obtained from the patient and unrelated healthy individuals, and HEK293 cells were cultured in Dulbecco's Modified Eagle Medium supplemented with 10% fetal bovine serum, 100 μg/ml streptomycin and 100 μg/ml penicillin in a humidified 5% CO_2_ incubator at 37°C. The cDNA fragment encoding human aconitase 2 (amino acid residues 1–780; GenBank NM_001098.3; generous gift from Dr. Agnès Rötig, Université Paris Descartes, Paris, France) was inserted into multicloning sites of the pCMV‐Myc vector (Clontech, Palo Alto, CA) to yield pCMV‐Myc‐hACO2 WT. Expression vectors for three human aconitase 2 mutants (p.Gly259Asp, p.Asp512Asn, and p.Gly666Ala) were made by site‐directed mutagenesis using the QuikChange Lightning Site‐Directed Mutagenesis Kit (Stratagene, La Jolla, CA) with pCMV‐Myc‐hACO2 WT as a template. Transient transfection of HEK293 cells with each expression vector was performed using polyethylenimine (PEI; PEI MAX, Polysciences, Inc. Warrington, PA) according to manufacturer's instructions, and the cells were subjected to the aconitase assay and Western blot analysis 48 hr after transfection. To investigate the effects of proteasome inhibition on the level of aconitase 2 in patient fibroblasts and HEK293 cells transfected with myc‐ACO2 constructs, MG132 (Sigma, St. Louis, MO) was added to the medium at a concentration of 5 μM. After incubation for 0, 4 or 8 hr, the cells were subjected to immunofluorescence analysis or Western blot analysis. An aconitase assay was performed using the Aconitase Assay Kit according to the manufacturer's directions (Cayman Chemical, Ann Arbor, MI). In HEK293 cells transfected with myc‐ACO2 constructs, the endogenous aconitase activity, measured from mock‐transfected cells, was subtracted from each value in order to estimate the net activities of myc‐ACO2 proteins. The endogenous aconitase activity of HEK293 cells was about 10 – 20% of the total aconitase activity of HEK293 cells expressing myc‐ACO2 WT. Immunofluorescence analysis and Western blot analysis, including densitometric quantification of each protein, were performed as previously described (Fukada, Nakayama, Mamiya, Yao, & Kawaguchi, 2016). The antibodies used in this study were commercially available and obtained from suppliers as follows: anti‐aconitase 1 (ab126595) and anti‐aconitase 2 (ab129069) were from Abcam (Cambridge, MA), anti‐ E1 subunit of pyruvate dehydrogenase (PDH E1α) (D‐6) (sc‐377092) and anti‐myc 9E10 (sc‐40) were from Santa Cruz Biotechnology (Santa Cruz, CA), and anti‐α–tubulin was from Cedarlane (Hornby, Ontario, Canada).

## 3 RESULTS

### 3.1 Clinical report

The patient was a Japanese girl born normally at 40 weeks weighing 3,082 g (−0.4 *SD*), with a height of 53 cm (+1.3 *SD*) and head circumference of 33 cm (+0.2 *SD*), as the second child of healthy, unrelated parents. She has an older sister without any developmental problems. Her first manifestation appeared at 9 days of age as downward eyeball displacement with fixed flexion posture of both elbows, suggesting focal seizures, but there were no abnormal findings on head magnetic resonance imaging (MRI) or electroencephalogram (EEG) at that time.

She was referred to Aichi Prefectural Colony Central Hospital at 6 monthsof age due to delayed motor development. At the initial visit, she showed poor voluntary movement and ataxia, and could not hold up her own head. Her body weight, height and head circumference were 7.7 kg (−0.2 *SD*), 67 cm (−1.2 *SD*) and 43 cm (−1.0 *SD*), respectively. On physical examination, she presented generalized hypotonia and hyporeflexia of the upper and lower limbs. Ophthalmologically, nystagmus and a poor reaction to photic stimulation were observed, but the ocular fundus was normal. She had no external malformations or dysmorphic facial features. Laboratory data, including lactate and pyruvate levels, and amino acid profiles in blood and cerebrospinal fluid, and the urine organic acid profile, were all normal.

**Figure 1:**
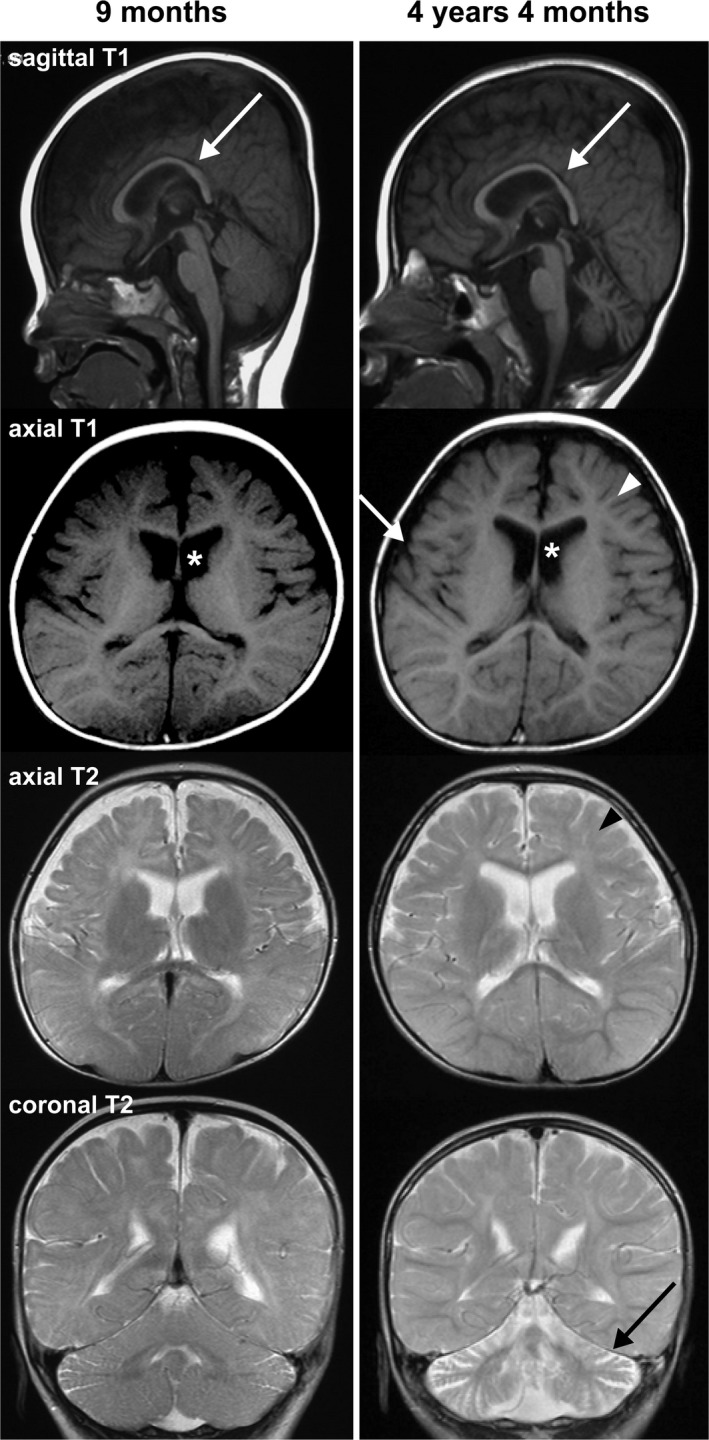
Brain magnetic resonance imaging examinations of the patient at 9 months and 4 years 4 months revealed progressive cerebral and cerebellar atrophy, and white matter abnormalities. T1‐weighted (sagittal and axial) and T2‐weighted (axial and coronal) images are shown. Malformation of the corpus callosum (sagittal T1, arrows), lateral ventricular enlargement (axial T1, asterisks), white matter abnormalities (axial T1 and 2, arrowheads), and cerebral (axial T1, arrow) and cerebellar (coronal T2, arrow) atrophy are marked in the figure

Thereafter, periodic brain MRI examinations were performed at 9 months, 2 years and 5 months, and 4 years and 4 months of age, and revealed progressive cerebellar and cerebral atrophy, enlargement of lateral ventricles and white matter abnormalities (Figure 1). In the auditory brainstem response (ABR) test performed at 2 years of age, waves III – VII were not detected even with 100 dB stimulation. At 3 years of age, she still had difficulty with head and trunk control and showed growth retardation: her body weight, height and head circumference were 8.8 kg (−2.7 *SD*), 88.5 cm (−1.1 *SD*), and 45.6 cm (−1.8 *SD*), respectively. After 3 years of age, the seizures were controlled with carbamazepine, and EEG analysis performed at 5 years of age showed multifocal spike discharges and spike‐and‐slow wave complexes. At 5 years of age, she had no improvement in motor function; she was bedridden and unable to sit in bed without support. She had no word or meaningful expressions. She died of pneumonia at 5 years of age.

### 3.2 Genetic analysis

Among the rare nonsynonymous variants identified by whole exome sequencing, we found two heterozygous mutations in *ACO2*, which were confirmed as compound heterozygous mutations using Sanger sequencing (Figure S1A). The c.1534G > A, p.Asp512Asn mutation transmitted from her mother was predicted to be “possibly damaging (score: 0.586)” by PolyPhen‐2 (Adzhubei et al., 2010) and “damaging” by SIFT (Kumar, Henikoff, & Ng, 2009). The c.1997G > C, p.Gly666Ala mutation transmitted from her father was predicted to be “probably damaging (score: 1.000)” by PolyPhen‐2 and “damaging” by SIFT. Asp512 and Gly666 are highly conserved from yeast to humans (Figure S1B), and both mutations were absent in our in‐house 575 Japanese controls. Both mutations were extremely rare because p.Gly666Ala was absent and p.Asp512Asn was only found in two out of 277,228 alleles in the gnomAD database (http://gnomad.broadinstitute.org/). According to X‐ray crystal structure analysis of bovine aconitase 2 (Lauble, Kennedy, Beinert, & Stout, 1992), Gly666 (corresponding to Gly639 of bovine aconitase 2) is located on the surface of a large cleft forming the pathway of the substrate to the active center, and is in the vicinity of Ser669 (corresponding to Ser642 of bovine aconitase 2), which is indispensable for catalytic activity. Asp512 is located on the molecular surface far from the active center (Robbins & Stout, 1989).

### 3.3 Biochemical analysis

**Figure 2:**
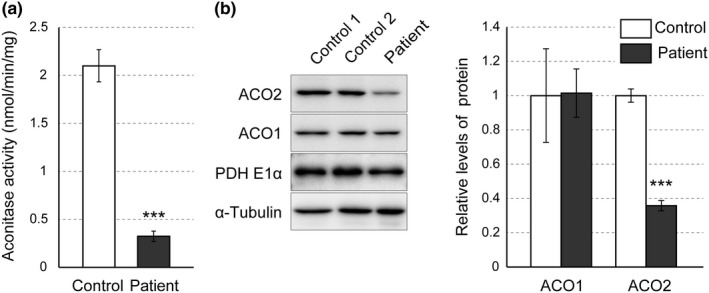
The aconitase activity and amount of aconitase 2 protein were both reduced in patient fibroblasts. (a) The aconitase activity in control and patient fibroblasts. Fibroblasts were lysed and cell extracts were assayed for aconitase activity. Data (expressed as nmol per minute per mg protein of cell extract) represent mean values ± *SD* from three independent experiments with triplicate measurements in each. The aconitase activity in patient fibroblasts was reduced to approximately 15% of that in control fibroblasts. (b) Western blot analysis of cell extracts from control and patient fibroblasts with antibodies against aconitase 2 (ACO2), aconitase 1 (ACO1), E1α subunit of pyruvate dehydrogenase (PDH E1α) and α‐tubulin. The signal of the patient ACO2 band was much weaker than those of the controls. The right graph shows the densitometric quantification of Western blot results. Data shown are the levels of aconitase 1 and aconitase 2 (normalized by α‐tubulin) in patient fibroblasts relative to those in controls, and are mean values ± *SD* from three independent experiments. ***p < 0.001 by the unpaired, two‐tailed Student's *t* test

**Figure 3:**
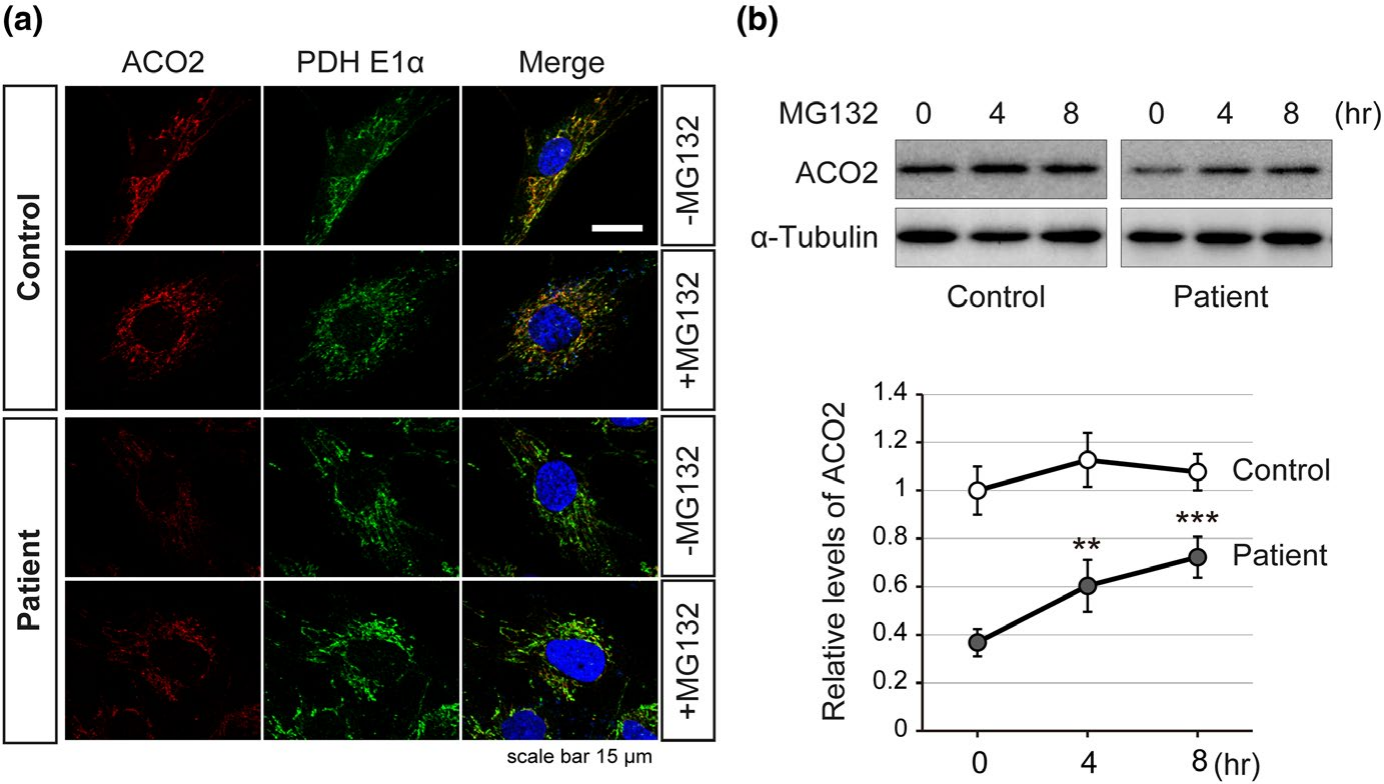
The reduced amount of aconitase 2 in patient fibroblasts was partially recovered by inhibiting proteasome activity. (a) Confocal immunofluorescence images showing subcellular localization of aconitase 2 (ACO2; red), E1α subunit of pyruvate dehydrogenase (PDH E1α; green) and nuclei (DAPI; blue) in control and patient fibroblasts treated for 0 or 8 hr with the proteasome inhibitor MG132 (20 μg/ml). PDH E1α was used as a mitochondrial marker. The aconitase 2 signals in the patient cells were very weak compared to those in the control (see −MG132 panels), but were clearly enhanced after MG132 treatment (see +MG132 panels). (b) Western blot analysis of aconitase 2 expression in fibroblasts treated with MG132 (20 μg/ml) for 0, 4 or 8 hr. The upper panel shows a representative Western blot, and the lower graph shows the densitometric quantification of Western blot results. Data represent aconitase 2 levels (normalized by α‐tubulin) relative to the control (0 hr), and are mean values ± *SD* from three independent experiments. **p < 0.01, ***p < 0.001 for 0 hr versus 4 or 8 hr (Patient) by the unpaired, two‐tailed Student's *t* test

In order to clarify the influence of *ACO2* mutations (p.[Asp512Asn;Gly666Ala]) on enzyme activity, we measured the aconitase activity of fibroblasts originating from the patient. The aconitase activity in patient fibroblasts was 0.32 ± 0.05 nmol min^‐1^ mg^‐1^, which was reduced to approximately 15% of that in control fibroblasts (2.10 ± 0.17 nmol min^‐1^ mg^‐1^) (Figure 2a). Western blot analysis revealed that the amount of aconitase 2 protein in patient fibroblasts was decreased to approximately 36% of that in controls (Figure 2b). In contrast, the expression levels of aconitase 1, a cytoplasmic aconitase, the E1 subunit of pyruvate dehydrogenase (PDH E1α), a marker of mitochondria, and α‐tubulin were comparable among the samples (Figure 1b). The decrease in aconitase 2 proteins in patient fibroblasts was also evident by immunocytochemistry (Figure 2a). The aconitase 2 signals (red), mostly colocalizing with mitochondrial marker signals (PDH E1α, green), were highly reduced in patient fibroblasts compared with control fibroblasts. The intensity, number and distribution of PDH E1α signals were comparable between the control and the patient samples (Figure 3a), indicating that the mitochondria were maintained in patient fibroblasts. Of note, the decrease in aconitase 2 signals in patient fibroblasts was partially restored by MG132, a proteasome inhibitor (Figure 3a,b). This suggests that the decrease in mutant aconitase 2 proteins was due to accelerated degradation by the proteasome system.

**Figure 4:**
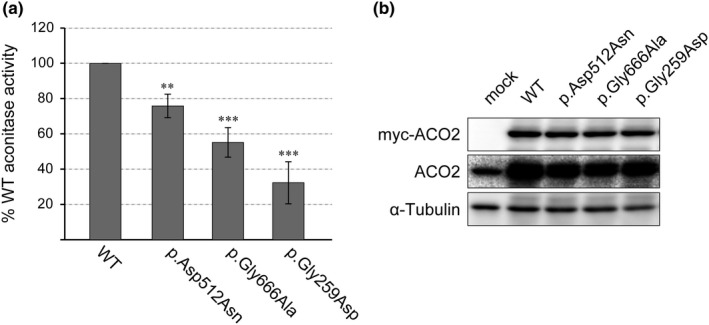
Comparison of aconitase activity of the wild‐type aconitase 2 and three mutants.(a) myc‐tagged aconitase 2, wild‐type (WT), and p.Asp512Asn, p.Gly666Ala and p.Gly259Asp mutants were each expressed in HEK293 cells, and cell extracts were assayed for aconitase activity. Values ± *SD* are shown as the percentage of WT in three independent experiments, each having triplicate assays. **p < 0.01, ***p < 0.001 for WT versus mutant aconitase 2, by the unpaired, two‐tailed Student's *t* test. (b) Western blot analysis of the cell extracts used in “A” with anti‐myc, ACO2 and α‐tubulin antibodies. The amounts of myc‐tagged aconitase 2 proteins (myc‐ACO2) used in the assay were all comparable

Next, we examined the respective effects of the p.Asp512Asn and p.Gly666Ala mutations on aconitase activity. For this, each myc‐tagged wild‐type or mutant aconitase 2 protein was expressed in HEK293 cells, and their activity was measured (Figure 4a). The aconitase activity of p.Asp512Asn and p.Gly666Ala mutants was approximately 76% and 55% of that of wild‐type, respectively. The activity of the p.Gly259Asp mutant, which was applied as a control and confirmed to have severely reduced aconitase activity in fibroblasts originating from homozygous patients (Metodiev et al., 2014), was approximately 32% of that of wild‐type. This supports the reliability of the data obtained by this assay using exogenously expressed aconitase 2 variants. The amounts of myc‐tagged aconitase 2 variants in the cell extracts used for the assay were all comparable. The destabilization of mutant aconitase 2 proteins (p.[Asp512Asn; Gly666Ala]) observed in patient cells, namely a decrease in the amount of mutant aconitase 2 (Figure 2b) and an increase in mutant aconitase 2 by proteasome inhibition (Figure 3), was not reproduced in experiments using HEK293 cells overexpressing mutant aconitase 2 (Figure 4b, data not shown). The amount of aconitase 2 proteins forcibly expressed in HEK293 cells is more than five times higher than that of endogenous aconitase 2 (Figure 4b, middle panel), which may overcome the degradation by the proteasome.

## 4 DISCUSSION

In this study, we identified compound heterozygous mutations in *ACO2* (p.[Asp512Asn; Gly666Ala]) in a patient with progressive cerebellar and cerebral atrophy, hypotonia, ataxia, seizure disorder, developmental delay, ophthalmological abnormalities and hearing loss. A p.[Asp512Asn; Gly666Ala] is a combination of novel missense mutations that has not been reported to date and causes a marked decrease in aconitase activity in patient cells. The pathogenicity of these mutations is evident because the symptoms are highly similar to those in previous reports of *ACO2* deficiency (Abela et al., 2017; Marelli et al., 2018; Metodiev et al., 2014; Sadat et al., 2016; Spiegel et al., 2012; Srivastava et al., 2017).

Including this report, the pathogenic *ACO2* mutations reported thus far are two homozygous mutations (17 patients from four families) and seven compound heterozygous mutations (nine patients from seven families). Table 1 summarizes the aconitase activity and aconitase 2 level in patient cells together with the main clinical features of all nine variants. Significant decreases in aconitase activity in patient cells were confirmed in all variants except two (p.[Gly620Asp; Gly683Val] and p.[Val364Ala; Lys776Asnfs*49], no information available), and the severity of symptoms is well‐correlated with the residual aconitase activity. Indeed, patients carrying p.[Leu74Val;Gly661Arg] mutations that resulted in the highest residual aconitase activity (60%–66%) developed no symptoms other than ophthalmological abnormalities, whereas patients carrying the homozygous p.Gly259Asp mutation that resulted in very low aconitase activity (5%) died at 57 or 61 days, although the detailed symptoms were unavailable (Metodiev et al., 2016). The changes in the aconitase 2 level in patient cells have been examined for five variants. There were no changes in two (p.Gly259Asp and p.[Arg607Cys; Pro712Leu]), and significant decreases were observed in three variants, including those in this study (p.[Asp512Asn; Gly666Ala], p.[Leu74Val; Gly661Arg] and p.[Lys736Asn; Lys776Asnfs*49]) (Metodiev et al., 2014). A decrease in the aconitase 2 level is a direct cause of reduced aconitase activity, leading to suppression of the TCA cycle.

**Table 1:**
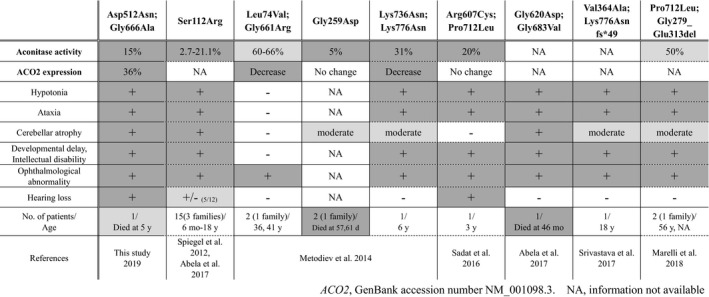
Clinical comparisons of reported patients with pathogenic variants in *ACO2*

We revealed that reduced aconitase activity in patient fibroblasts harboring p.[Asp512Asn; Gly666Ala] mutations (Figure 2a) was caused by the combination of two factors. The first is the decrease in the aconitase 2 protein amount in patient fibroblasts (36% of that of the control) (Figure 2b), due to accelerated degradation by proteasomes (Figure 3). The second is the moderate but significant reduction of aconitase activity in individual mutants that is p.Asp512Asn (76%) and p.Gly666Ala (55%) (Figure 4). As in our case, when the mutant aconitase 2 protein has a certain degree of enzyme activity and its degradation is considered to be the main cause of the low cellular aconitase activity, restoration of the protein level by proteasome inhibition is expected to be effective to recover aconitase activity. However, due to the limited amount of patient cells, we were unable to confirm this possibility, but it is worth considering in similar cases.

The pathological mutations of *ACO2* ultimately cause mitochondrial dysfunction. Indeed, many of the symptoms of *ACO2* deficiency are common to mitochondrial disease and other TCA cycle enzymopathies, including deficiency of α‐ketoglutarate dehydrogenase (EC 1.2.4.2; Odièvre et al., 2005), succinate dehydrogenase (EC 1.3.5.1; Bourgeron et al., 1995), fumarate hydratase (EC 4.2.1.2; Bourgeron et al., 1994) and succinate‐coenzyme A ligase (EC 6.2.1.5; Elpeleg et al., 2005; Ostergaard et al., 2007). However, it should be noted that the elevation of lactate in the serum, cerebrospinal fluid and urine, which is usually detected in mitochondrial diseases, is not observed in *ACO2* deficiency. Therefore, to diagnose *ACO2* deficiency, sequence analysis of*ACO2* and direct measurement of aconitase activity in patient cells are needed. In this regard, quantification of cis‐aconitate, an intermediate product of the aconitase reaction, may be useful to diagnose *ACO2* deficiency because a significant reduction of cis‐aconitate in the plasma of *ACO2* patients (p.Ser112Arg, p.[Gly620Asp; Gly683Val]) was found by liquid chromatography and mass spectrometry (LC‐MS)‐based metabolomics analysis (Abela et al., 2017).

Our knowledge of the symptoms of *ACO2* deficiency is increasing. In order to explore the possibility of medical intervention for *ACO2* deficiency, the etiology and exact pathogenic mechanisms must be clarified by analyzing the biological effects of *ACO2* mutations.

## CONFLICT OF INTEREST

None.
